# Regulation of amino acid transporters in the mammary gland from late pregnancy to peak lactation in the sow

**DOI:** 10.1186/s40104-018-0250-4

**Published:** 2018-04-08

**Authors:** Fang Chen, Shihai Zhang, Zixiao Deng, Qiqi Zhou, Lin Cheng, Sung Woo Kim, Jun Chen, Wutai Guan

**Affiliations:** 1 Department of Animal Science, South China Agricultural University, Guangzhou, 510642 China; 2 National Engineering Research Center for Breeding Swine Industry, Guangzhou, 510642 China; 3 Department of Animal Science, North Carolina State University, Raleigh, NC 27695 USA

**Keywords:** Amino acid transporter, Lactation, Mammary epithelial cell, Milk protein, Sow

## Abstract

**Background:**

Milk protein is crucial for milk quality in sows and health of newborn piglets. Plasma amino acids (AA) in sows are important precursors for milk protein synthesis in the mammary gland. In order to study the regulation of AA transported in sow mammary glands and possible underlying mechanisms, we measured the expression of genes coding for milk proteins, AA transporter expressions, and plasma AA concentrations in sows at three different physiological stages (D-17, D1 and D17 of lactation), and then further investigated the regulation of AA transport across the cell membrane by adaptive mechanisms using pig mammary epithelial cells (PMEC) as an in vitro model. PMEC were cultured in DMEM:F12 with 4 amino acid concentrations (0 × AA complex, 1 × AA complex, 5 × AA complex, and 25 × AA complex). Classes of AA complexes evaluated in this study included neutral AAs (*L*-Ala + *L*-Ser + *L*-Cys), acidic AAs (*L*-Asp, *L*-Glu) and neutral + basic AAs (*L*-Ala + *L*-Ser + *L*-Cys + *L*-Lys).

**Results:**

Our results indicated that mRNA expression of genes coding for milk protein (αs1-casein, αs2-casein, β-casein and κ-casein) increased significantly with the advance of physiological stage (*P* < 0.05), and plasma concentrations of most AAs including threonine, serine, glutamate, alanine, valine, cysteine, methionine, isoleucine and tyrosine were greater at D1 of lactation compared with D-17 and D17 of lactation (*P* < 0.05). Additionally, protein and gene expressions of AA transporters including excitatory AA transporter 3 (EAAT3), alanine/serine/cysteine/threonine transporter (ASCT1) and sodium-coupled neutral AA transporter 1 (SNAT2) were greater in lactating sow mammary glands compared with sow mammary glands in late pregnancy (*P* < 0.05). The mRNA expressions of *SLC38A2*, *SLC1A1, SLC6A14* increased significantly in the cell mediums supplemented with 5 × and 25 × of AA complexes compared with those cells cultured in DMEM/F12 cell medium (*P* < 0.05). The mRNA expressions of *SLC38A, SLC1A4*, and *SLC6A14* also increased in EBSS cell medium compared to DMEM/F12. However, only mRNA expression of *SLC38A* decreased when AA complex was added into EBSS (*P* < 0.05).

**Conclusion:**

AA transportation was positively regulated in sow mammary glands with the advance of physiological stage from late pregnancy to peak of lactation and AA transporters in PMECs were adaptively regulated by changed AA concentrations.

## Background

The mammary gland undergoes extensive morphological, structural, and functional changes from pregnancy to lactation in order to provide optimal nutritional support for offspring with different nutrient requirements at different developmental stages [1–4]. Studies in several species indicated that there may be species-specific changes in milk composition in addition to the changes in amount of milk secretion at different stages of lactation, corresponding to growth phases of the young offspring [5]. Research in dairy cows and mice discussed possible mechanisms of this phenomenon, and reported that there was a rapid adaptation in mammary glands at different stages of development with altered expression of several genes involved in lactogenesis [6, 7]. However, there are limited reports in the peer reviewed literature focusing on regulation of milk protein synthesis in the sow, a multifetal mammal with high daily milk production.

Our lab previously screened 1,524 genes differentially expressed at three different physiological stages (D-17, D1, D17 of lactation) using Affymetrix Porcine GeneChip and Gene Ontology analysis. Results indicated that most of the regulated genes were involved in transportation of nutrients and biosynthetic processes related to nutrients synthesis in mammary tissue [8]. Considering that protein, fatty acids, and lactose are the major components in sow milk and essential for growth and health of neonatal pigs, we further investigated regulatory mechanisms of fatty acid and lactose synthesis in mammary tissue in two previous published papers [9, 10]. Milk proteins are of importance during the entirety of lactation to support growth and health of nursing piglets and the changes in milk protein contents at different stages of lactation are more pronounced compared to fatty acids and lactose [11]. Therefore, understanding mechanisms of milk protein synthesis and their regulation in pig mammary glands is important for development of technology to enhance growth and health of nursing piglets.

The mammary gland has a large demand for AAs to meet the requirements for milk protein synthesis during lactation and the availability of these AAs to the mammary system is critical for optimizing milk production [12]. It is well documented that intracellular availability of AAs is controlled by coordinated activity of AA transport systems and their respective carrier proteins, which are located on the cellular membrane and are responsible for channeling AAs from the arterial blood across the cell membrane [13, 14]. However, the regulation mechanism of transporter systems in sow mammary glands at different physiological stages of mammary glands with changed milk protein content are unknown.

Therefore, the objectives of this study were to 1) investigate the regulation of amino acid transporters in sow mammary glands at 3 different physiological stages (D-17, D1, and D17 of lactation), and 2) evaluate regulatory mechanisms of AA transporters under conditions with varied concentrations of AAs using PMECs as an in vitro model.

## Methods

### Animal management and tissue sample collection

Six multiparous (four to six parities) Large White sows on D90 of pregnancy were selected randomly from the Huizhou Swine Breeding Center and were fed and managed by established procedures. Sows were housed in individual feeding stalls with free access to water and feed. The diets were mainly based on corn and soybean meal containing nutrients meeting or exceeding NRC (2012) requirements (Table 1).

**Table 1:** Ingredients of the sow diets (air-dry basis)

Ingredients, %	Late stage of pregnancy	Lactation period
Corn	65.60	62.30
Soybean meal	22.0	23.0
Soybean oil	1.00	3.00
Wheat bran	8.0	5.0
Fish meal	0.0	3.0
Limestone	0.9	1.0
Calcium phosphate	1.2	1.3
Salt	0.3	0.4
Vitamin premix^a^	0.5	0.5
Mineral premix^b^	0.5	0.5
Total	100.0	100.0
Nutrition standards, %		
DE, MJ/kg	13.45	13.87
CP	16.05	17.71
CF	3.75	3.40
EE	4.12	5.76
Ca	0.86	0.84
P	0.63	0.68
AP	0.41	0.44
Lys	0.86	1.02

^a^Provided the following per kilogram of diet: 2,400 IU of vitamin A, 3,000 IU of vitamin D_3_, 60 mg of vitamin E, 5 mg of vitamin K, 5 mg of vitamin B_1_, 12.5 mg of vitamin B_2_, 24 mg of pantothenic acid, 50 mg of niacin, 5 mg of vitamin B_6_, 0.037 mg of vitamin B_12_, 2.2 mg of folacin, 0.1 mg of biotin

^b^Provided the following per kilogram of diet: 8 mg of Cu, 60 mg of Fe, 35 mg of Mn, 65 mg of Zn, 0.35 mg of I, 0.3 mg of Se

Mammary glands were categorized by their anatomical locations according to the standard described by Kim et al. [15]. Approximately 1 g of mammary tissue was excised using the surgical method reported by Manjarin et al. [16]. Briefly, sows were anesthetized by intramuscular injection of mixed anesthetics that contained 500 mg Shutai (Chongbiwei Biological Technology Inc., Beijing, China) and 350 mg ketamine (Agricultural Bureau of Guangzhou City supply, Guangzhou, China) dissolved in 0.9% sodium chloride. After disinfecting the mammary glands with 75% ethanol, a 2-cm incision was made vertical to the plica lateralis, aligned with the nipple, and approximately 5 cm dorsal to the perimeter of the nipple areola. About 0.8 g mammary tissue was excised with a scalpel in a circular motion. The incision was closed with simple interrupted sutures using braided silk (Jinhuan, Inc., Shanghai, China). Sows received an intramuscular injection of ampicillin sodium (Jiacheng Technology, Inc., Zhuhai, China) at a dose of 10 mg/kg immediately after biopsy and then 24 and 48 h later. Sutures were removed 7 d after biopsy. Tissue samples were immediately frozen in liquid nitrogen. In total, 18 mammary gland samples were collected. All procedures were conducted following the protocols approved by the Committee for the Care and Use of Experimental Animals at South China Agricultural University.

### Milk collection and milk composition analysis

Colostrum samples were collected on the day of parturition, within 4 h of the birth of the first piglet. Milk samples were collected on D17 post-farrowing following the intramuscular administration of 2 mL oxytocin (10 IU/mL; NVS, Basel, CH) before percutaneous biopsies. The samples were stored without preservative at − 70 °C until analyzed for milk composition. An automatic milk composition analyzer (ML4AC, Beijing, China) was used to measure the contents of lactose, protein, fat and solids-not-fat components in the milk.

### Cell culture to evaluate effect of AA concentrations on gene expression of AA transporters in PMEC

PMEC cells used in this study were previously separated and characterized from mammary glands of lactating sows in our lab. Briefly, a 1-cm^3^ sample of mammary tissue from a lactating sow was cut using surgical scissors and then the tissue pieces were dissociated by gentle agitation at 37 °C for 12 h. After filtration through stainless steel mesh (80-mesh) to remove dissociated tissue and debris, the cells were collected by centrifugation at 800×*g* for 3 min at 25 °C. The isolated PMEC cells were cultured in DMEM/F12 (GIBCO) containing 10% fetal calf serum, insulin (5 μg/mL), hydrocortisone (5 μg/mL), ITS (5 μg/mL), IGF-1 (10 ng/mL), EGF (10 ng/mL), 50 μg/mL gentamicin and PSN antibiotics. The attached cells were washed 3 times with D-PBS containing PSN antibiotics after a 24-h cultivation, and then cell medium was added for continued culturing. Immunohistochemistry and RT-PCR were further performed to characterize these separated mammary epithelial cells by protein expression of cytokeratin-18 (an intermediate filament specific for epithelial cells), and gene expression of β-casein (a specific marker for mammary epithelial cells), β-lactoglobulin and α-lactalbumin.

Cells were grown in DMEM:F12 (GIBCO, Thermo Fisher Scientific, Waltham, MA, USA), containing 5% (*w*/*v*) heat-inactivated fetal calf serum (GIBCO, Thermo Fisher Scientific, Waltham, MA, USA), insulin (bovine, 5 μg/mL, Sigma, St. Louis, Mo, USA), gentamicin (50 μg/mL, Sigma, St. Louis, MO, USA) and combined antibiotics (penicillin, 100 IU/mL and streptomycin 0.1 mg/mL, Biological Industries) at 37 °C under humidified 95% air/5% CO_2_. When the cells were approximately 80% confluent, they were seeded in 6-well culture plates (5 × 10^5^ cells/well) and then were assigned to 4 concentrations of AA complexes in each of 2 mediums for each of 3 classes of AA complexes. The mammary epithelial cells were allowed to attach overnight in 6-well culture plates and then AA complexes (including 0 × AA complex, 1 × AA complex, 5 × AA complex, and 25 × AA complex) were added into cell medium for 4 h treatment for subsequent real time-PCR analysis. There were three classes of AA complexes evaluated in this study, which included neutral AAs (*L*-Ala + *L*-Ser + *L*-Cys), acidic AAs (*L*-Asp, *L*-Glu) and neutral + basic AAs (*L*-Ala + *L*-Ser + *L*-Cys + *L*-Lys). Individual AAs were purchased from Sigma Company (St. Louis, MO, USA) and the concentrations of AA in 1 × AA complexes were compounded according to their concentrations in the DMEM:F12 cell medium as follows: neutral AAs (*L*-Ala, 0.05 mmol/L/; *L*-Ser 0.25 mmol/L; *L*-Cys 0.1 mmol/L), acidic AAs (*L*-Asp 0.05 mmol/L; *L*-Glu, 0.05 mmol/L); neutral + basic AAs *(L*-Ala, 0.05 mmol/L; *L*-Ser 0.25 mmol/L; *L*-Cys 0.1 mmol/L; *L*-Lys, 0.499 mmol/L). The AA concentrations in 5 × AA complexes were compounded as follows: neutral AAs (*L*-Ala, 0.25 mmol/L; *L*-Ser 1.25 mmol/L; *L*-Cys 0.5 mmol/L), acidic AAs (*L*-Asp 0.25 mmol/L; *L*-Glu, 0.25 mmol/L); neutral + basic AAs *(L*-Ala, 0.25 mmol/L; *L*-Ser 1.25 mmol/L; *L*-Cys 0.5 mmol/L; *L*-Lys, 2.495 mmol/L), and the AA concentrations in 25 × AA complexes were compounded as follows: neutral AAs (*L*-Ala, 1.25 mmol/L; *L*-Ser 6.25 mmol/L; *L*-Cys 2.5 mmol/L), acidic AAs (*L*-Asp 1.25 mmol/L; *L*-Glu, 1.25 mmol/L); neutral + basic AAs *(L*-Ala, 1.25 mmol/L; *L*-Ser 6.25 mmol/L; *L*-Cys 2.5 mmol/L; *L*-Lys, 12.475 mmol/L). AA depletion was achieved by changing the cell medium to Earle’s Balanced Salt solution (EBSS, Sigma, St. Louis, MO, USA) containing insulin, hydrocortisone and prolactin for 4 h and then AA complexes (including 0 × AA complex, 0.04 × AA complex, 5 × AA complex, and 25 × AA complex) were subsequently added into EBSS. The components of EBSS were as following: CaCl_2_▪2H_2_O, 0.265 g/L; MgSO_4_, 0.09767 g/L, KCl, 0.4 g/L; NaHCO_3_, 8.2 g/L; NaCl, 6.8 g/L; NaH_2_PO_4_, 0.122 g/L; *D*-Glucose, 1.0 g/L; Phenol RedCNa, 0.011 g/L. The cells were harvested after 4 h for mRNA determination using Real-time PCR. Gene expressions of *SLC38A2* and *SLC1A4* were measured in those cells treated with neutral AA complexes, *SLC1A1* was measured in those cells treated with acidic AA complex, and *SLC6A14* was measured in those cells treated with neutral + basic AAs complex.

### Measurement of the plasma AAs concentration

Plasma AA concentrations of sows were determined by automatic AA analyzer (LP-8900, Hitachi, Tokyo, Japan). Plasma samples (400 μL) were deproteinized with 1,200 μL of 10% sulfosalicylic acid and the supernatant was assayed for AA composition. S-(2-amino-ethyl)-*L*-cysteine was used as an internal standard.

### Real-time PCR

Total RNA was isolated from 100 mg tissue samples using TRIZOL reagent (Invitrogen, Carlsbad, CA, USA) according to the manufacturer’s instructions. The RNA quality was confirmed by calculating the OD 260/280 ratio via a spectrophotometer, and the integrity of the RNA was verified by agarose gel electrophoresis. The qPCR was performed in an ABI Prism 7500 Sequence Detection System using SYBR Green Real-time PCR Master Mix (Toyobo, Osaka, Japan) in a final volume of 20 μL after cDNA synthesis which was performed using a PrimeScript RT reagent kit with gDNA eraser (Takara, Dalian, China) according to the protocol described by Shu et al. [8]. All the primer sequences for PCR are shown in Table 2 and Table 3. The thermal cycling conditions of qPCR reactions were as follows: 95 °C for 1 min followed by 40 cycles of denaturation at 95 °C for 15 s, annealing at 59 °C for 15 s, and extension at 72 °C for 40 s. A melting curve analysis was also performed for each gene to confirm the production of a single specific amplification product. The amplification efficiency of each gene was counted and the relative expression level for each gene was calculated using the equation described previously [17]: R_0_, _T_/R_0_, _R_ = (1 + E_R_)^Ct,R^/(1 + E_T_) ^Ct,T^ (E = amplification efficiency, R = reference gene, T = target gene, E_R =_ amplification efficiency of reference gene, E_T_ = amplification efficiency of target gene, C_t,R_ = threshold cycles for reference gene, C_t,T_ = threshold cycles for target gene).

**Table 2:** Primers for the genes of milk protein

Gene name	Accession number	Primers (5'→3')	Size, bp
*CSN1S1*	NM_001004029	F: ACAAATGAGGACAAGCATACCC	175
		R: GAGGGATGTTGGTGAATAATGG	
*CSN1S2*	NM_001004030	F: GAAGTGGGATATGCCAGCAG	148
		R: CCTGGAGATATTGGGGGAAC	
*CSN2*	NM_214434	F: TCATCCTTGCCTGCTTCGT	367
		R: GGCATTCCTTTACGCTTGG	
*CSN3*	NM_001004026	F: GCCTATCCTGGCATTAACACTG	286
		R: TGGGTAGACATTTGGCTGGTC	

*Abbreviation*: *CSN1S1* αS1-casein, *CSN1S2* αS2-casein, *CSN2* β-casein, *CSN3* κ-casein

**Table 3:** Primers for the genes of AA transporters

Gene name	Gene accession	Primers sequence (5′→3′)	Size, bp
*SLC1A1*	NM_001164649	F: TCCACTCCATTGTTATTCTGCC	172
		R: TTGTCCACCTGGTTCTTCTCTTC	
*SLC1A2*	HSU03505	F: AGCAGGTGGAAGTGCGAATG	160
		R: CGAAGAAGCCCTCCACACACT	
*SLC1A3*	BC037310	F: TCACGCAGTCATCGTCTTGC	171
		R: TTGTCCACGCCATTGTTCTC	
*SLC1A4*	XM_003125088	F: AGACCTCTCTTTGATCCTGGC	190
		R: TGTTTCCTCCTCTGATTTGCA	
*SLC1A5*	XM_003127237	F: AAGGAGTCGGTTCTGTGATGG	108
		R: TAAAAGTCGGCGAGGGTGA	
*SLC3A1*	EU587017	F: TGCATGACATTGTCCGCAG	140
		R: TTGGATAAAAGGCAACCCG	
*SLC3A2*	XM_003353809	F: TGAACAGCAGCGTGACTGTG	162
		R: ACTGGCGGATGTAGGAGAAGA	
*SLC6A14*	GQ387269	F: TCCAGAAGCTCTAGCCCAACT	190
		R: CAAAACCAAGCAGCAACCC	
*SLC6A15*	NM_018057	F: GCTTCTTGGGCTTTGTGATG	232
		R: GGTGAGTCCGTGCTTGTTTC	
*SLC7A1*	NM_001012613	F: GCCTGAGAGCAAGACCAAAC	166
		R: GCCGTAGCCGAAGTAGATGA	
*SLC7A2*	EU155140	F: GCCCCAGAATCAGCAAAAAGTA	97
		F: GATGCTGAAGGCTGGCAAAA	
*SLC7A5*	NM_003486	F: CTCTTCCTGATCGCCGTCTC	165
		R: CTTCTGACACAGGACGGTCGT	
*SLC7A6*	NM_003983	F: CTGCCGCCTGCATGTGT	78
		R: TGTGCCCCACTTGACATAGG	
*SLC7A7*	EU047705	F: TTTGGTTCCCAAGGTTGCA	95
		R: GCAGCTTCCTGGCATTGC	
*SLC7A8*	AF135830	F: TCGCTGTGACTTTTGGAGAGA	112
		R: CGGGAGGAGGTGAAGAGG	
*SLC7A10*	XM_003127014	F: TGGACCCTCGAAAGAACCTAC	216
		R: TGATCCCTCCGAAAGTGGA	
*SLC7A11*	AB271957	F: AATGTGGCCTACTTCACGACC	193
		R: CCTCCCGAGATGCAACATAGA	
*SLC38A1*	XM_003355629	F: AAGAACCTGGGCTATCTCGG	138
		R: TGTTGCGTTAGGACTCGTTG	
*SLC38A2*	NM_018976	F: GTTACCTTTGGTGATCCAGGC	96
		R: ACCAATGACACCAGCAGAACC	
*SLC38A4*	XM_003355630	F:GTTCTTTGCCTTCACTCACTA	186
		R: GACCCAAGCCTCCAGATT	
*MRPL39*	AY610067	F: CAAAAGAGAACCTACATTCCTTCACA	100
		R: TCTAATGCCACTTTTGCTTCAACT	

### Western blot

Mammary tissue samples were homogenized in RIPA lysis buffer (Beyotime, Nanjing, China). Extracted protein samples were heated in water at 100 °C for 5 min and separated by electrophoresis (Bio-Rad, Richmond, CA) in 12% SDS-PAGE gel before being electroblotted (Bio-Rad) onto a polyvinylidene fluoride membrane (Millipore, Billerica, MA). After polyvinylidene fluoride membranes were blotted with Tris buffer containing 5% fat-free dry milk and 0.05% Tween-20 (TBST; 0.05% Tween 20, 100 mmol/L of Tris-HCl, and 150 mmol/L of NaCl, pH 7.5) for 1 h at 25 °C, they were rinsed in TBST 4 times and incubated overnight at 4 °C with primary antibodies: EAAT3 (Sigma Aldriche, sc-25658), ASCT1(Sigma Aldriche, sc-50801), and SNAT2 (Sigma Aldriche, sc-166366) with ratios of 1:1,000, 1:800, and 1:1,000 respectively. Rabbit IgG (5 μg/mL; Sigma) was used as the primary antibody for controls. After washing, membranes were incubated with secondary antibody (Amersham Biosciences). The chemiluminescent signal was detected by using ECL reagents (Beyotime, Nanjing, China), and bands were quantified by Image Processing Software (Image Pro Plus6.0).

### Statistical analysis

Milk, plasma amino acid, and mRNA expression data were analyzed by one-way analysis of variance (ANOVA) using SPSS 17.0 with physiological stage as the fixed effect in the model and variation among sows within physiological stage as the random error. Cell culture data were also analyzed as a one-way classification with AA concentration/medium combination (treatment) as a fixed effect and variation among replicates within treatment as random error. The cell culture experiment was performed with six replicates per treatment combination. Data are shown as means ± SEM. *P* < 0.05 was considered to be statistically significant and *P* < 0.01 was considered to be highly statistically significant.

## Results

### Component analysis of colostrum and milk

It is clear that there were significant differences in lactose, milk fat and milk protein between colostrum and milk (Table 4). The concentrations of lactose and milk fat were greater in milk compared to colostrum (*P* < 0.05), while milk protein concentration was less in milk at D17 of lactation (*P* < 0.05). There was little evidence of a difference in solids-not-fat (*P* > 0.1).

**Table 4:** The component analysis of colostrum and milk

Item, %	Colostrum	Milk	*P*-value
Lactose	9.38 ± 0.19^A^	11.29 ± 0.37^B^	0.00
Milk fat	2.61 ± 0.32^B^	9.45 ± 1.59^A^	0.00
Milk protein	8.06 ± 0.23^A^	3.55 ± 0.39^B^	0.00
Solids-not-fat	19.32 ± 0.56	17.86 ± 1.04	0.27

Note: All data shown as means ± SEM, values with different capital letter superscripts differ (*P* < 0.01)

### The mRNA expression of milk proteins (αs1-casein, αs2-casein, β-casein and κ-casein) in mammary tissue at different physiological stages

The gene expressions of these four important milk proteins increased markedly with physiological stage with the least expression at D-17, intermediate expression at D1 and the greatest expression at D17 of lactation (*P* < 0.05) (Table 5).

**Table 5:** The relative mRNA abundance of *CSN1S1*, *CSN2*, *CSN1S2* and *CSN3*

Gene	D-17	D1	D17	*P* -value
*CSN1S1*	69.56 ± 10.55^A^	497.22 ± 24.23^B^	1179.49 ± 74.96^C^	0.00
*CSN1S2*	7.45 ± 1.46^A^	224.86 ± 52.09^B^	1673.83 ± 190.20^C^	0.00
*CSN2*	1.10 ± 0.17^A^	69.31 ± 11.44^B^	170.75 ± 19.44^C^	0.00
*CSN3*	12.73 ± 1.55^A^	394.18 ± 49.17^B^	1193.37 ± 132.85^C^	0.00

Note: All data shown as means ± SEM, values with different capital letter superscripts differ (*P* < 0.01)

### Concentration of AAs in sow plasma at different physiological stages

The concentrations of AAs in sow plasma differed among the three different physiological stages (including D-17, D1 and D17 of lactation) (Table 6. Several AA concentrations including threonine, serine, glutamate, alanine, valine, cysteine, methionine, isoleucine, tyrosine, lysine and histidine were significantly higher on the first day of lactation than D-17 and D17 of lactation (*P* < 0.05). In addition, the concentration of phenylalanine was significantly higher during lactation compared with pregnancy (*P* < 0.05), and the concentrations of aspartic acid and glutamate were greater on D1 compared to D-17 (*P* < 0.05). There was little evidence of differences in leucine, ornithine and arginine at different physiological stages.

**Table 6:** Comparison of free AA in sow blood plasma at different physiological stages

AA, μmol/L	D − 17	D 1	D 17	*P*-value
Thr	21.03 ± 2.00^A^	47.44 ± 4.31^B^	15.26 ± 1.28^A^	0.00
Ser	15.99 ± 1.73^a^	22.27 ± 2.14^b^	14.42 ± 1.09^a^	0.02
Glu	22.35 ± 0.65^a^	27.19 ± 3.33^b^	23.15 ± 1.56^ab^	0.03
Gly	70.11 ± 3.48^B^	47.34 ± 1.49^Aa^	56.40 ± 1.26^Ab^	0.00
Ala	34.30 ± 3.00^A^	56.75 ± 5.44^B^	25.91 ± 0.86^A^	0.00
Val	1.77 ± 0.11^A^	5.14 ± 0.68^B^	2.63 ± 0.26^A^	0.00
Cys	13.26 ± 0.99^A^	18.71 ± 1.60^B^	11.20 ± 0.67^A^	0.00
Met	27.15 ± 1.22^A^	37.87 ± 3.98^B^	26.54 ± 0.85^A^	0.01
Ile	20.14 ± 1.33^A^	30.82 ± 3.73^B^	21.32 ± 0.81^A^	0.01
Leu	18.33 ± 0.44	22.46 ± 2.68	17.92 ± 1.85	0.24
Tyr	22.08 ± 1.49^Aa^	32.05 ± 2.95^B^	28.18 ± 0.92^Ab^	0.01
Phe	12.74 ± 1.03^Aa^	18.25 ± 2.36^Ab^	20.01 ± 1.09^B^	0.03
Orn	6.43 ± 0.69	8.59 ± 1.46	6.68 ± 0.61	0.27
Lys	18.86 ± 2.08^A^	34.41 ± 2.77^B^	20.31 ± 2.17^A^	0.00
His	8.75 ± 1.09^a^	13.16 ± 1.62^b^	10.36 ± 1.49^a^	0.02
Arg	42.28 ± 3.61	40.86 ± 3.61	42.01 ± 5.85	0.31
Asp	1.03 ± 0.17^a^	1.96 ± 0.21^b^	1.44 ± 0. 15^ab^	0.04

Note: All data shown as means ± SEM, values with the same lower case letter superscripts do not differ (*P* > 0.05), with different lower case letter superscripts differ (*P* < 0.05), and with different capital letter superscripts differ (*P* < 0.01)

### The mRNA expressions of AA transporters at different stages (D-17, D1 and D17) in sow mammary glands

We estimated the gene expressions of 19 AA transporters (including anionic, cationic, neutral, neutral cationic and heavy subunit AA transporters) related to milk protein synthesis at 3 physiological stages (D-17, D1 and D17 of lactation) in this study.

**Fig. 1:**
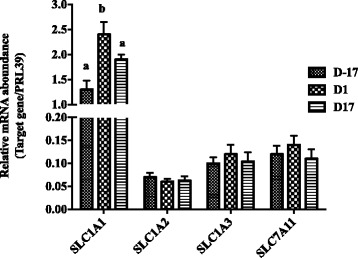
The mRNA abundance of anionic AA transporters at different stages in porcine mammary gland. Gene expression of anionic AA transporters in sow mammary gland tissues at D-17, D1 and D17 of lactation. All data are shown as means ± SEM. In the histogram of the same gene, values with different lower case letter superscripts differ (*P* < 0.05)

**Fig. 2:**
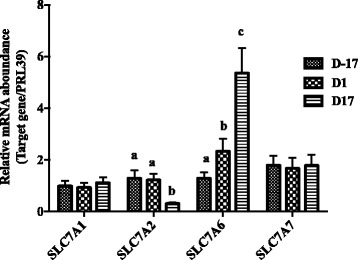
The mRNA abundance of cationic AA transporters at different stages in porcine mammary gland. Gene expression of cationic AA transporters in sow mammary gland tissues at D-17, D1 and D17 of lactation. All data are shown as means ± SEM. In the histogram of the same gene, values with different lower case letter superscripts differ (*P* < 0.05)

**Fig. 3:**
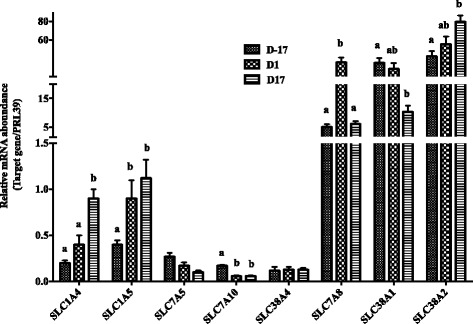
The mRNA abundance of neutral AA transporters at different stages in porcine mammary gland. Gene expression of neutral AA transporters in sow mammary gland tissues at D-17, D1 and D17 of lactation. All data are shown as means ± SEM. In the histogram of the same gene, values with different lower case letter superscripts differ (*P* < 0.05)

**Fig. 4:**
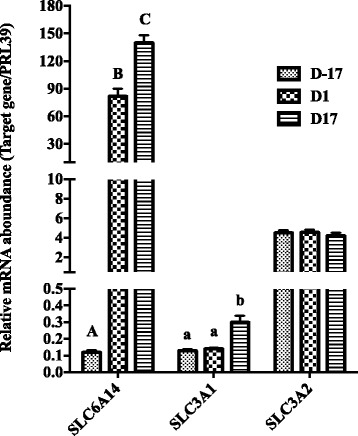
The mRNA abundance of neutral cationic AA transporters and Heavy subunit (HSHAT) AA transporters at different stages in porcine mammary gland. Gene expression of neutral cationic AA transporters and Heavy HSHAT AA transporters in sow mammary gland tissues at D-17, D1 and D17 of lactation. All data are shown as means ± SEM. In the histogram of the same gene, values with different lower case letter superscripts differ (*P* < 0.05); those with different capital letter superscripts differ (*P* < 0.01)

The gene *SLC1A1* had the greatest expression among 4 anionic AA transporters (including *SLC1A1*, *SLC1A2*, *SLC1A3* and *SLC7A11*), and its expression increased significantly at D1, at which day the sows transited from pregnancy to lactation, and then decreased at D17 of lactation (Fig. 1, *P* < 0.05). We estimated gene expressions of 4 cationic AA transporters (*SLC7A1*, *SLC7A2*, *SLC7A6*, *SLC7A7*) (Fig. 2). Expression of *SLC7A2* decreased at D17 postpartum compared with D-17 and D1 (*P* < 0.05), while *SLC7A6* significantly increased along with the physiological stage (*P* < 0.05). Gene expressions of 8 neutral AA transporters were estimated, including *SLC1A4, SLC1A5, SLC7A5, SLC7A10, SLC38A4, SLC7A8, SLC38A1*, and *SLC38A2* (Fig. 3). Expressions of *SLC1A4, SLC1A5* and *SLC38A2* increased with time of sample (*P* < 0.05), while the expressions of *SLC7A10* and *SLC38A1* decreased at D17 of lactation compared with D-17 (*P* < 0.05), and expression of *SLC7A8* was greatest on D1 compared to D-17 and D17 (*P* < 0.05). Gene expressions of neutral cationic AA transporters and heavy subunit AA transporters *(SLC6A14, SLC3A1, SLC3A2*) at three different physiological stages are shown in Fig. 4. Expression of SLC6A14 increased over 70 fold on D1 postpartum compared to pregnancy (*P* < 0.05), and then continued to increase at a lesser rate to D17 of lactation (*P* < 0.05). There was little evidence of differences in *SLC3A2* at the 3 different physiological stages, but *SLC3A1* increased at D17 of lactation, compared with the other two time points (*P* < 0.05).

### Protein expression of ASCT1 (SLC1A4), EAAT3 (SLC1A1) and SNAT2 (SLC38A2) at different stages (D-17, D1 and D17) in porcine mammary glands

**Fig. 5:**
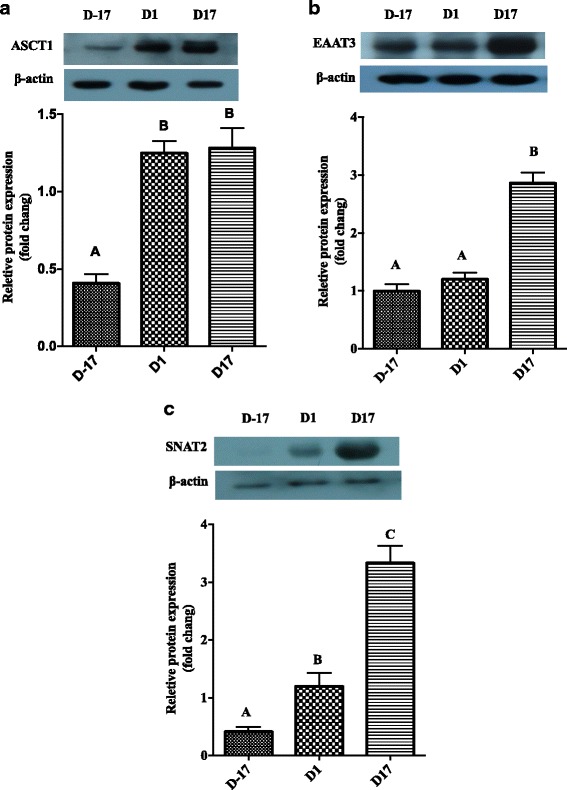
Protein abundance of ASCT1, EAAT3 and SNAT2 at different stages in porcine mammary gland. Protein expression of AA transporters, including ASCT1 (**a**), EAAT3 (**b**) and SNAT2 (**c**) in sow mammary gland tissues at D-17, D1 and D17 of lactation. All data are shown as means ± SEM. In the histogram of the same gene, values with different lower case letter superscripts differ (*P* < 0.05); those with different capital letter superscripts differ (*P* < 0.01)

We estimated protein expression of three AA transporters to confirm if protein expression was consistent with mRNA expression (Fig. 5). Protein expression of *ASCT1* was greater on both days postpartum compared with D-17, and protein expression of *EAAT3* was greatest at D17 of lactation compared to D-17 and D1 (*P* < 0.05). Protein expression of SNAT2 increased from D-17 to D1 and continued to increase to D17 (*P* < 0.05).

### The adaptive regulation of AA transporters in PMEC

In order to determine response of PMECs to changes in AA concentrations, we added different concentrations of AAs into cell medium and estimated AA transporter gene expressions using Real-time PCR. We also used EBSS as an AA-deficient cell-culturing medium and estimated AA transporter gene expressions after addition of different concentrations of AA complexes.

**Fig. 6:**
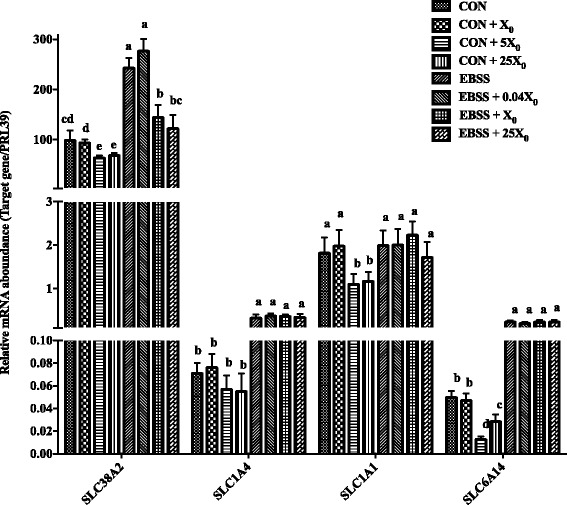
The gene expression of SLC38A2, SLC1A4, SLC1A1, SLC6A14 in PMEC cells cultured with different concentration of AAs. Cells were assigned to 4 concentrations of AA complex in each of 2 mediums for each of 3 classes of AA complexes (Neutral AAs complex: *L*-Ala + *L*-Ser + *L*-Cys; Acidic AA complex: *L*-Asp + *L*-Glu; Neutral + basic AAs complex: *L*-Ala + *L*-Ser + *L*-Cys + *L*-Lys). Gene expression of SLC38A2 and SLC1A4 were detected in those cells treated with neutral AA complexes, SLC1A1 were detected in those cells treated with acidic AA complex, and SLC6A14 were detected in those cells treated with Neutral + basic AAs complex. All data are shown as means ± SEM. In the histogram of the same gene, values with different lower case letter superscripts differ (*P* < 0.05)

Gene expressions for *SLC38A* and *SLC1A4* were detected in those cells treated with the neutral AA complex (*L*-Ala, *L*-Ser and *L*-Cys), gene expression for *SCL1A1* was detected in those cells treated with the acidic AA complex (*L*-Asp and *L*-Glu), and gene expression for SLC6A14 was detected in those cells treated with the neutral + basic AA complex (*L*-Ala, *L*-Ser, *L*-Cys and *L*-Lys) (Fig. 6).

Gene expression of *SLC1A4* was minimally affected when the amount of neutral AA complex was increased from 1 × to 25 ×, but the expression of *SLC38A2* decreased when the AA complex concentration was increased to 5 × compared with the control *(P* < 0.05). The expressions of both *SLC38A2* and *SLC1A4* were increased when AAs were eliminated from cell culturing medium, and *SLC38A2* gene expression decreased when AA complex was re-added into EBSS (*P* < 0.05). However, there was little change in *SLC1A4* expression when AA complexes were re-added into EBSS.

The gene expression of *SCL1A1* in PMECs decreased significantly in the DMEM:F12 cell medium which was supplemented with 5 × and 25 × acidic AA complex (*P* < 0.05), but there was little effect when the basal medium was changed to EBSS, and even when acidic AA complex was re-added into EBSS medium. We observed the same trend when we treated the PMECs using neutral + basic AA complexes. The expression of *SLC6A14* was lower in the groups with 5 × and 25 × AA complex addition (*P* < 0.05), but there was little difference between EBSS group and EBSS + AA complex group.

## Discussion

It is well known that milk proteins are critical component of milk quality for mammals due to their important roles in newborn animal development and health [18]. Although lactation is a continuous process of milk secretion, there are two different phases of lactation: colostral phase (the colostrum contains large amounts of immunoglobulins and other immune defense proteins), and the mature secretion phases with much greater milk production supporting the rapid growth of the newborn [19]. In the present study, we analyzed milk components in colostrum (D1) and milk at D17 of lactation and the results showed that protein concentration in colostrum is significantly higher compared with milk at D17 postpartum, which are consistent with previous studies [20]. Because of the increased milk yield but decreased protein concentration on D17 of lactation from first day of lactation to the peak day, the changes of total milk protein synthesis were not clear. Therefore, we further estimated mRNA expression of genes coding for primary milk proteins in mammary tissue (αs1-casein, αs2-casein, β-casein and κ-casein) to determine the regulation of milk protein synthesis at different physiological stages, both pre- and postpartum. Results demonstrated that mRNA expression of those genes coding for milk protein increased markedly from late pregnancy to peak milk production, implying more milk protein synthesis at peak lactation.

The porcine mammary gland has a large demand for free AAs to meet its high rate of milk production during lactation since more than 90% of milk protein is synthesized de novo in mammary tissue from the free AAs taken up from circulation [21]. Therefore, AAs provided to mammary glands are essential precursors for milk protein synthesis and their availability is a key limiting factor for milk protein production [22, 23]. We determined the concentrations of free AAs in sow plasma, both pre- and postpartum, and the results showed that most AAs, including threonine, serine, alanine, valine, cysteine, methionine, isoisoleucine, tyrosine, phenylalanine, lysine, etc., increased significantly when the sow transitioned from pregnancy to lactation at D1. However, this rise in plasma AAs from late pregnancy to D1 postpartum was generally not maintained to D17 of lactation (day of peak lactation) despite estimates of milk protein synthesis being greatest at D17. It is a common phenomenon that sows, especially those with more than 12 piglets/litter, lose significant body weight during lactation because body tissues are catabolized to provide substrates for extensive milk synthesis in mammary glands [24]. Previously, several studies reported that AAs were released from muscle tissues through protein degradation and the degradation rate increased with the advance of lactation [25–27]. In our study, the increases in AA concentration at D1 of lactation might be attributed to protein degradation of sow body tissue to meet the requirement of milk protein synthesis and lower concentration of AAs at D17 of lactation might be a consequence of higher milk protein synthesis with more AAs uptake from sow plasma AA at peak lactation.

Milk protein synthesis is not only determined by sow plasma AA availability, but is also controlled by transporter efficiency of AAs in the mammary glands [28, 29]. Guan et al. reported that mammary net uptake of some individual AAs (e.g., lysine) is a crucial rate-limiting step for the synthesis of milk protein in vivo [30] and Shennan et al. reported that the uptake of AAs from blood into the mammary glands was enhanced to sustain the synthesis of milk proteins during lactation [31]. In some species, such as cattle and mice, genes involved in transport processes also have been reported to be upregulated with stimulation of lactation [7]. AA uptake by the mammary gland is performed by different transporters located on the plasma membrane of the mammary epithelial cells. Generally, these systems can be subdivided into neutral, cationic, anionic, and others [32], some of which transport multiple AAs into and out of the cells as well [33]. We estimated the gene expressions of up to 8 neutral AA transporters, including *SLC1A4, SLC1A5, SLC7A5, SLC7A10, SLC38A4, SLC7A8, SLC38A1* and *SLC38A2* at the three different physiological stages, and we found that expressions of *SLC1A4*, *SLC1A5* and *SLC38A2* were relatively greater compared with other transporters, implying their dominant roles in AA transport during milk protein synthesis process. A number of studies reported that the *SLC38A2* gene plays an important role in AA transport in mammary glands. For example, Tovar et al. reported that the expression of *SLC38A2* increased significantly from late pregnancy to the start of lactation and then continue to the peak of lactation [34]. In our study, the gene of *SLC38A2* increased significantly with the advance of lactation with maximal expression at D17 of lactation, suggesting that the efficiency of AA transport was enhanced to meet the demands of milk protein synthesis. Additionally, we also found the expressions of *SLC1A4* and *SLC1A5*, the other two important neutral AA transporters, were increased significantly with the advance of lactation, which was consistent with the expression of *SLC38A2*. The gene expressions of both *SLC1A1* (anionic AA transporter) and *SLC7A6* (cationic AA transporter) also significantly increased with the advance of lactation, which result was consistent with neutral AA transporters. Our present results suggest that increased milk protein synthesis and net AA uptake across the mammary gland with the advance of lactation from late pregnancy to peak lactation might be attributed to the enhanced expression of AA transporters.

It has been reported that AA transporters are regulated by several hormones, including insulin, glucagon, glucocorticoids growth hormone, and thyroid hormone etc. [35]. However, the role of a second type of AA transport regulation termed “adaptive regulation” is still unclear. In this process, the activity of AA transporters increases when cells are exposed to conditions of AA shortage or starvation (adaptive depression), while decreases when cells are exposed to conditions of abundant substrate AA (adaptive repression), as reported by Gazzol et al. [36]. It has been previously reported that mRNA abundance of AA transporters could be modulated by dietary protein intake and mammary AA uptake increased linearly with increasing dietary protein, suggesting a coordinated regulation between AA transporters and concentration of AAs to meet the demands for milk yield [37, 38]. In our study, although the changes in expression of each AA transporter with increased supplemental AA concentration differed, gene expressions of *SLC38A, SCL1A1* and *SLC6A14* declined significantly when we added up to 25 × supplemental AA complex into PMEC mediums. This implies that sufficient duration and higher concentration of supplemental AAs would inhibit the expression of corresponding AA transporters to adaptively limit the uptake of AAs into PMECs. The gene expression of *SLC38A4* increased significantly 4 h after AA elimination from the cell medium, then decreased to the level close to control group when substrate AAs were introduced back into the medium. It appears that adaptive regulation links transport activity and availability of AAs in the cell environment by affecting the expression of AA transporters in PMECs. It is noteworthy that the expressions of *SLC1A4* and *SLC6A14* increased rapidly under the condition with AA deficiency to ensure adequate uptake of AAs into PMECs, and the transporters continued to maintain increased expression even after reintroducing substrate AA into the cell medium. We speculate that these two transporters are less sensitive to AA concentration fluctuations and their expression in PMECs appears to require longer times to adjust to variations in AA concentrations, which possible mechanisms will be investigated in future studies. Furthermore, we also found that the gene expression of *SCL1A1* showed higher stability evidenced by relatively less change in the AA supplemented groups and also no change in the AA starvation and shortage groups, implying different regulatory abilities of different transporters in PMECs.

## Conclusions

In summary, AA transportation was positively regulated in sow mammary glands with the advance of physiological stage from late pregnancy to peak of lactation. Additionally, AA transporters in PMEC showed adaptive regulation with changes in AA concentrations of PMECs mediums. This novel finding is the first to report the expression and regulation mechanism of AA transporters at different physiological stages in the sow, providing basic information for the mechanism of milk protein synthesis in pigs and the potential to improve milk quality for swine industry.

## Abbreviations

AA: Amino acid
ASCT1: Alanine/serine/cysteine/threonine transporter 1
EAAT3: Excitatory AA transporter 3
EBSS: Earle’s balanced salt solution
PMEC: Porcine mammary epithelial cell
SNAT2: Sodium-coupled neutral AA transporter 1
